# OUTCOMES OF A PILOT WELLNESS PROGRAM FOR LOW-INCOME OLDER ADULTS IN INDEPENDENT LIVING: A MIXED METHODS STUDY

**DOI:** 10.1093/geroni/igae098.3877

**Published:** 2024-12-31

**Authors:** Khalid Yusuf, Isabelle Erb, Joan Tunningley, Victor Ronis-Tobin

**Affiliations:** Xavier University, Cincinnati, Ohio, United States; Xavier University, Cincinnati, Ohio, United States; Xavier University, Cincinnati, Ohio, United States; Xavier University, Cincinnati, Ohio, United States

## Abstract

By 2030, one-fifth of U.S. residents will be of retirement age, leading to increased demand for residential services for older adults (OAs). Effective wellness programming is crucial to support the physical and psychological well-being of low-income residents, yet there is limited evidence to guide such program development and evaluation. This pilot study details the first phase of a program development initiative in two independent living communities in Southwest Ohio managed by Episcopal Retirement Services (ERS). A mixed-methods approach was used to inform program design. Participants were 57 residents, with an average age of 75.71 years (SD = 7.75); 89.5% were female, and 59.6% were Black or African American. Most participants (86%) reported incomes below $25,000, and 52.6% were divorced. Baseline engagement levels were low (M = 19.82, SD = 6.92), as measured by the Social Engagement and Activities Questionnaire (SEAQ). SEAQ scores did not correlate with program attendance; however, significant positive correlations were observed between going outside the home and both the number of friends (r(55) =.380, p =.004) and having friends within the residence (r(55) =.314, p =.018). Focus group analysis (n = 13) identified eight themes: Holistic Engagement, Activities for Social Connection, Participation Challenges, Personalization Desires, Impact of Isolation, Male Engagement Issues, Strategies for Boosting Engagement, and Self-Improvement Aspirations. These findings emphasize the need for wellness programs that address physical health, foster social connections, and promote personal growth. Active resident-staff collaboration in the development and maintenance of such programming is crucial for success.

